# London Correspondence

**Published:** 1867-03

**Authors:** 


### SELECTIONS.

From the Southern Journal of the Medical Sciences, New Orleans, La.

### FOREIGN CORRESPONDENCE.

*London*, November, 1866.

The marvellously rapid development of the Arts and Sciences in the present day must make every one who is in any way connected with either of them, take stock occasionally of the condition of his own department, and of the comparative progress which is being made in it from year to year. The surgeon who will first reduce himself to the proper degree of humility, by remembering what remains to be done, may fairly turn from such a review as I have proposed with reassurance, and some amount of complacency. For not only can he point to such achievements of modern times as the discovery of general and local anæsthesia, ovariotomy, and the excision of diseased joints, but there is also to be observed a steady and sterling advance in the execution of more every day work, which shows itself, not only in the improvement in the results of treatment, and in the fact that many conditions which formerly ranked incurable, may now be referred to as curable; but, also, on the greater humanity of the means employed, and in the greater care which is taken to secure the ease and comfort of the sufferers. Much progress of this latter kind has lately been made in the management of fractures, in illustration of which I may refer to the treatment of fractures of the femur; of the patella; and of the bones of the leg, as now conducted in Mr. Paget's wards, at St. Bartholomew's Hospital. I have great pleasure in noting the method for fracture of the thigh first, for it is one which was exhibited to Mr. Paget's class, some months ago, by Dr. Smith, of the Southern States of America. It is that which consists in swinging the limb by means of an iron wire frame fixed along its upper aspect. As your readers are doubtless quite familiar with the apparatus, I need say only this much in description of it, which may suffice to allow you to identify it. Experience of its use here has brought it into high favor. It is very easily adjusted, and probably the surgeon who should be called upon to conduct the treatment of a fractured thigh, where he was thrown on his own resources for an apparatus, would find this easier to construct out of rough materials than any other that is in use. Patients express themselves strongly as to the comfort with which they can bear it; and the gratification expressed by one or two patients who, after having borne the long splint through part of the treatment, had this adjusted instead, was very significant of the relief they felt. It will be necessary to have a larger experience of its use than we have already gained, before any denfiite opinion as to its value can be pronounced; but I may give shortly the particulars of two cases in which its merits are well illustrated. The first was that of a woman, thin, withered and pale, and looking seventy, though she said she was only sixty-four, who was admitted under Mr. Paget's care, with fracture of the neck of the femur. Her general health was so poor that it seemed dangerous to subject her to any rigidly fixed position, or to any long confinement to bed. The limb was placed in Dr. Smith's "sling." From the first, everything went on well. After the first few days she was able to sit up in bed to take her food, or merely for a change of position. She slept well, and complained of no discomfort. At the end of six weeks the limb was taken out of the sling, and it was found that the fracture was well united; and the muscles had so well retained their power that she could raise her leg from the bed.

The second case was that of a young woman æt. twenty-six, who was admitted for necrosis of the lower third of the shaft of the right femur. Her health was much reduced by prolonged suppuration, and the want of good food. After a previous unsuccessful attempt to extract the sequestrum, which lay enclosed in a thick casing of new bone, Mr. Paget, by removing the necessary amount of the outer wall of the femur, having first divided the biceps, and turned it back, was enabled to pull out the sequestrum. The operation entailed a very considerable wound, and the condition of the parts was such as to make extensive suppuration in the inter-muscular spaces of the thigh very likely, unless a free exit for discharge could be provided. It seemed of the greatest importance that no check towards recovery should be allowed, for the patient was greatly wasted, and had little strength left. The thigh was "slung" so that the wound was dependent; and as this was cone-shaped, with its apex at the femur, the discharge, instead of lodging, ran directly out, and fell into a vessel containing a disinfecting fluid. The wound thus required no other treatment than to be syringed out twice a day. The woman's recovery was very rapid: she quickly regained flesh and color; and the local mischief was soon repaired by granulation. A more admirably adapted apparatus than the "sling" proved to be in this instance, could scarcely be imagined.

Here I may mention in parenthesis, that the Liston's long splint, which has for many years been looked upon by English surgeons as the sheet anchor in the treatment both of fractures of the femur and diseases of the hip-joint, is falling into disuse. For the past three years it has scarcely been used at all in the hospital for sick children, for disease of the hip, the substitute being continuous extension applied by means of the "strapping-stirrup" and a weight. This method, which is recommended by its simplicty and comfort, is found to give the most satisfactory results.

It has been, till recently, the custom in England, in the treatment of transverse fractures of the patella, to use a bed jointed in the middle of its length, so that the patient's limb could be raised and flexed upon the pelvis, nearly to a right angle, in order, as it was said, to relax the muscles in front of the thigh, and so to prevent the separation of the upper from the lower fragment of the patella. This apparatus, which obliged the patient to remain in what was equivalent to the sitting posture, all through the period of treatment, was most irksome. About three years ago, Mr. Paget, at St. Bartholomew's Hospital, discarded this method, and allowed the limb to be outstretched horizontally upon the bed; and from that time to the present, all the cases of transverse fracture that have come under his care, have been so treated. Many surgeons have now adopted the plan, and it is found that the results obtained are fully as good as those secured by the older method. The case against raising the limb has been thus ably put by Mr. Jonathan Hutchinson, in the London Hospital Reports, 1865, page 338. "The muscles, when the limb is at rest, are not contracted, but quite flaccid. Examine the patella of a person in bed, and you will find it loose and moveable; the muscles are not acting in it in the least. Make the same examination in a case of fracture of the patella, after the first spasm has subsided, and when the patient is not voluntarily contracting his quadriceps, and you will find the muscle wholly relaxed. My second objection is, that even if the quadriceps were in constant action, which I assert it is not, you could not diminish the distance between its attachment and origin in a material degree by lifting the limb. All that part of the muscle which arises from the femur, (the crureus and the two vasti) will not be in the least altered. Lift the limb as high as you like, you move both origin and insertion together, and equally, and cannot possibly alter their relative positions. The rectus, it is true, arises above the hip joint, but is so close to the latter that the length of the muscle is but very little modified by flexion of the thigh. My chief reason, however, is obtained from clinical experience. Over and over again, we have proved by experiment in lifting and lowering the limb, in cases of transverse fracture, that position does not, in the slightest degree, prevent the bringing down of the upper fragment." In the large majority of the cases, the treatment, both by Mr. Paget and Mr. Hutchinson, has consisted solely in leaving the limb horizontal, without any kind of appliance for bringing the fragments into contact. In the few instances in which any such have been used, very slight, if any, good has seemed to follow.

Mr. Paget thus treats fractures of the leg: The limb is placed on an iron back-splint, which has a rectangular foot piece, and which reaches four or five inches beyond the knee. This splint is fixed in position by two caps of gutta percha, softened in hot water, and moulded, the one over the front of the foot, and the other over the knee; then two wooden side splints are adjusted: these are fixed by being buckled together across the sole, with which their lower extremities are exactly on a level, and by web-straps which encircle them halfway up the leg, and again above the knee. The limb is then "slung." In cases of compound fractures, the side splints are "interrupted" opposite the wound, should this chance to be on the lateral aspect of the limb. It will be noticed that the peculiarities of this method (which are also its advantages) are, first, its extreme simplicity; second, the absence of bandaging. There is no circular constriction of the limb, and the course of events at the seat of injury can be accurately known at any period of treatment; and which is an advantage, although a minor one, the skin can be sponged, and thus kept clean, and free from that troublesome itching which comes of confined perspiration. It is now a common practice in England to "sling" the upper extremity in cases of excision of the elbow, or of severe fracture, or of any serious disease; and the freedom of movement which it allows conduces much to the comfort of the patient. It is not an exaggeration to say that the "swinging" of limbs is one of the greatest improvements which, has of late years, beeh made in the mechanics of surgery.

As dislocations are so kindred a subject to fractures, it is right to mention the one point about them, which has, of late, been brought prominently forward—their reduction, in the case of the hip and shoulder joints, by "manipulation," instead of by the old method of extension. Mr. Nunneley, of Leeds, in a letter to the British Medical Journal, Oct. 20th, announced that in the practice of his colleagues and himself, at the Leeds Infirmary, in the last few years, out of twenty-one dislocations of the hip, manipulation was tried in all but one: it succeeded in fourteen, and failed in seven. In these seven, who were afterwards subject to the pulleys, three were reduced at the first attempt; one failed in a first attempt, but succeeded, after considerable difficulty, in a second; three failed altogether; and in another, though it was thought the bone was reduced, subsequently it was again found to be displaced, and could not be permanently reduced—making four cases in which, doubtless, fracture of the acetabulum, or of the neck of the bone, accompanied dislocation, and prevented reduction." This surgeon considers that the most important condition to be insured, when manipulation is to be tried, is a relaxed, but not perfectly helpless, flaccid, uncontractile state of the muscles; as it is by contractions of the muscles which are attached near the head of the dislocated bone, that reduction is mainly accomplished. This relaxed condition of the muscles is often present directly after a dislocation has occurred, and when the patient is in a state either of syncope or of shock; but when it is not present it should be induced by chloroform. Mr. Nunneley's paper immediately drew forth letters from many of the London, and some of the provincial hospitals, which proved that the method by "manipulation" was in very general use among English surgeons. Mr. John Birkett, of Guy's Hospital, bears the following testimony to its efficacy in his own hands. "Six dislocations of the femur on the dorsum ilii; five into the sciatic notch; four into the foramen ovale, and one on the ramus of the pubes, were reduced by manipulation (while the patients were under chloroform) without the aid of pulleys, during the last eighteen years."

This method of reducing dislocations, so much safer, so much less violent, and as the results above referred to show, so successful, is a great improvement on the old method by extension. I am sure English surgeons most willingly acknowledge their obligation to Dr. Reid, of Rochester, to whom is attributed the honor of having introduced it as a legitimate plan.

Desormeaux's endoscope has lately been brought prominently under notice, by Mr. Heath, of the Westminster Hospital, who has sent his experience of its use to the Lancet. I may briefly say that the instrument consists of a farafin lamp, enclosed for convenience in a wooden case, from which the light is conducted through a lens on to a perforated mirror, set at an angle in a cylinder. An eye piece is adapted to one end of this cylinder, and to the other a screw, by which the various tubes that are to be introduced into the several outlets of the body may be attached. Mr. Heath has "three tubes for the uretha, corresponding with No. twelve, ten and eight of the catheter guage, each measuring eight inches in length, and being provided with an aperture on one side, near the outer end, through which a wire carrying cotton wool may be introduced into the interior of the tube, and be made to reach its extremity, so as to wipe away any discharge from the surface under examination, or to apply medications to it. A large tube, of similar make, is very efficient for examining the rectum; and a tube, with a piece of glass at the end, can be used for examining the interior of the bladder, the glass preventing the entrance of wire into the tube." The best position in which to place the patient for examining the urethra is the recumbent one, on a table or sofa of good height, with the knees bent over and slightly separated. The surgeon then introduces the largest tube which the meatus will admit, and passes it gently until he meets with an obstruction, or finds, from the direction of the instrument, that he has entered the membranous urethra, when it is well to pause, lest on withdrawing the plug (which much facilitates the introduction, as with a vaginal speculum) a gush of urine take place. The tube is now attached to the tube of the endoscope, which is placed horizontally, and the light being adjusted, the operator proceeds to look through, when he will see the surface of the urethra, at the end of the tube, shutting down upon it with perfect regularity, if healthy; and by slowly withdrawing the instrument, with the eye still fixed at the cylinder, he will be able to note the various modifications which the urethra may have undergone in its whole length, if necessary, after examining the prostatic portion. By means of the endoscope, Mr. Heath could make out the "granular urethritis" of Desormeaux, which keeps up a constant slight discharge, and gives such trouble both to the patient and the surgeon. It was found "in various parts of the urethra, sometimes confined to a small spot, at others spreading for some distance; its most favorite spot being about the bulbous portion of the urethra." This granular condition, which may lead to a true organic stricture, is best described by comparing it with the condition of "granular eyelids." Organic stricture can be accurately made out by the endoscope, and it would probably facilitate the diagnosis of urethral chancre and urinary fistulæ. Marked relief followed, in many cases, the local application of nitrate of silver, through the tube of the endoscope. Mr. Heath says that in cases of organic stricture, he has succeeded in introducing a wire, through the endoscopic tube, into the contracted orifice, and has then passed over the wire an elastic catheter, which he has thus guided through the stricture: thus demonstrating the possibility of relieving the bladder in a case of difficult, and to ordinary methods of treatment, impermeable stricture. Mr. Henry Thompson, whose knowledge of, and whose skill in treating all diseased conditions of the urethra and bladder, are such as make his opinion on such a point of the greatest weight, writes that he "came to the conclusion, after many trials of it, that Desormeaux's endoscope was of small value in practice, as regards diseases of the urethra and bladder." * * * * "True it discloses a granular condition of the urethra, and enables the surgeon to apply an agent to the exact spot, but heretofore it was known that such conditions existed most commonly in the bulbous portion of the urethra; that the site of the diseased part was fairly appreciable by its undue sensibility when an instrument was passed over it, and that cure often followed the application of a solution of nitrate of silver to the sensative spot." And as regards stricture, he says: "I doubt the power of the endoscope to reveal the orifice of a stricture through which a good surgeon has been unable, with care and attention, to pass an instrument. I have long believed, with Mr. Syme, that a stricture which admits the passage of urine outwards in any quantity, will admit, to gentle and careful manipulation, the passage of an instrument inwards to the bladder." * * * * And "minute objects, such as the orifice of a small stricture, must be very difficult to find, for Dr. Cruise confesses that he cannot even detect (with the endoscope) the verumontanum, or the orifices of the ejaculatory ducts in the healthy urethra." Mr. Thompson objects to the use of the endoscope on account of its collision with "the really valuable maxim, which I implicitly believe to underlie all success in the surgical treatment of urethral and vesical diseases; namely, to diminish as much as possible, all sources of mechanical irritation. * * * * Every year I am more and more convinced of the value of this maxim, and more and more act upon its indications. It is this which has led me to perform lithotrity, invariably, without preliminary injections, and commonly without any subsequent one; and also limit the length of the sitting to one, or at most to two minutes. Lastly, in the treatment of stricture, to employ the softest and most flexible instrument, instead of the inflexible and metalic, in direct opposition to my earlier convictions, and to the special traditions of my school."

There are a very large number of surgeons who would completely endorse Mr. Thompson's opinions on all the points referred to. Nevertheless, there are doubtless cases in which the endoscope has its value.

Mr. Henry Lee has published the particulars of a case of aneurism, which he cured by acupressure, a method which he believes will prove very valuable in many instances of this disease. His patient, a boy of nineteen, was wounded in the lower part of the left popliteal space, with a sharp knife. Bleeding was very free, and recurred after the lapse of some days, and a tumor was found in the popliteal space of the size of a chestnut. This had all the features of an aneurism. "A long, slender needle, previously made for the purpose of acupressure, was introduced immediately to the outside of, and above the tumor, which was at the same time pressed inward by the point of the finger. The needle was made to penetrate deeply into the popliteal space, its point was then turned inward and brought out immediately behind the internal tuberosity of the tibia." The pulsation in the tumor stopped immediately after the needle was introduced; but the pulsation in the posterior tibial artery, in the lower part of the leg could be still distinctly felt. The aneurism was considered to be on one of the large branches of the popliteal, not on this vessel itself. The needle was removed on the sixth day. The patient recovered without a bad symptom.

Your readers are probably aware that Mr. J. Baker Brown (London) is in the habit of performing "clitoridectomy;" or, in simpler phrase, cutting off the clitoris, in a variety of nervous diseases in women, such as hysteria, epilepsy, and insanity, in the belief that these diseases very commonly depend on the habit of masturbation, and because, he says, he has found "clitoridectomy" effect a cure. The accusation which this doctrine involves against the sex, and the questionable physiology on which the alleged cure depends, have, from its first announcement, caused the operation to be regarded with much suspicion. This feeling has at length found expression, in a letter to the Lancet, by Dr. Charles West, whose opinion always commands the highest respect among the profession in England, and whose book on "The Diseases of Women," if I mistake not, is well known in America. As the matter has attracted much notice here, I will extract the main part of the letter. Dr. West says:

"1st. Having for the past twenty-five years seen more of the diseases of children, and young persons of both sexes, than most members of my profession, and as much as most, of the diseases of women at all ages, I believe that masturbation is much rarer in girls and women than in our own sex.

"2d. I believe the injurious *physical* effects of habitual masturbation to be the same as those of excessive sexual indulgence, and no other. The special physical harm done by masturbation, I believe to be due to the fact that it can be indulged in at a much earlier age than sexual intercouse, and can be practiced with much greater frequency.

"3d. But, nevertheless, I have not, in the whole of my practice, seen convulsions, epilepsy, or idiocy induced by masturbation in any child of either sex, a statement I need scarcely add, widely different from the general idea that epileptics or idiots may, and not seldom, do masturbate. Neither have I seen any instance in which hysteria, epilepsy, or insanity in women after puberty, was due to masturbation as its efficient cause.

"4th. I *know*, and I can appeal with confidence to the knowledge of many members of the medical profession, that of the alleged causes of hysteria, epilepsy, insanity, and other nervous diseases of women, by excision of the clitoris, a very large number were not permanent. I further *know* that in several instances, one of which, seen by me in consultation with Mr. Paget, is related at page 663 of my lectures, very mischievous results have followed it.

"5th. While I believe the removal of the clitoris, in cases of hysteria, epilepsy, insanity, or other diseases of women, to be a proceeding theoretically based on erroneous physiology, and practically followed by no such results as warrant its frequent performance, I regard it as completely unjustifiable when done for the alleged relief ef dysuria, or of painful defœcation; for the cure of amenorrhœa, or for the mitigation of the symptoms of uterine displacement or disease. (In all these conditions Mr. Brown has used it, as he reports, successfully.)

"7th. I consider that public attempts to excite the attention of non-medical persons, and especially of women, to the subject of self-abuse, in the female sex, are likely to injure society, and to bring discredit on the medical profession.

"8th. I believe that few members of the profession will dissent from the opinion that the removal of the clitoris without the cognizance of the patient and her friends, without full explanation of the nature of the proceeding, and without the concurrence of some other practitioner, selected by the patient, or her friends, is in the highest degree improper, and calls for the strongest reprobation."

I think it right to inform American surgeons, who may have heard of the practice of "clitoridectomy" in England, what is thought of it here. Dr. West's opinions may certainly be accepted as very faithfully representing those of a very vast majority of English practitioners.

The novelty of the past two months is the injection of acetic acid into the substance of cancers, in order to secure their removal by absorption. The plan is brought forward by Dr. Broadbent, a physician who is thought highly of in London. It suggested itself to his mind in consequence of the known solvent powers of this acid upon cancer cells, as seen under the microscope. Dr. Broadbent recommends that about forty drops of a mixture of one part of acetic acid with five or six parts of distilled water, should be injected into the cancer; the point of the syringe being thrust (subcutaneously) into different parts of its substance during the operation, so that the agent is well disseminated through it. The injected is repeated at intervals, varying from five to ten days, or longer, according to its effects. The method is now being extensively tried, and many cases are reported in which large cancers have been completely and almost painlessly removed. Its true value, however, can by no means be pronounced upon at present.

Although cholera has occupied much attention, during its late prevalence here, I have not ventured to enter upon so purely medical a subject. I think, however, I may say, without doing any one injustice, that no great advance has at present been made in its treatment; at all events, in many neighborhoods, the mortality among those attacked by it, has been as high as it is recorded to have been in previous outbreaks.

